# The Front-End Readout as an Encoder IC for Magneto-Resistive Linear Scale Sensors

**DOI:** 10.3390/s16091416

**Published:** 2016-09-02

**Authors:** Trong-Hieu Tran, Paul Chang-Po Chao, Ping-Chieh Chien

**Affiliations:** Department of Electrical Engineering, National Chiao Tung University, Hsinchu 200, Taiwan; tronghieu@ctu.edu.vn (T.-H.T.); james4252002@hotmail.com.tw (P.-C.C.)

**Keywords:** magnetic linear scales, encoder readout IC, programmable gain amplifiers (PGAs), successive approximation register (SAR) analog-to-digital converters (ADCs)

## Abstract

This study proposes a front-end readout circuit as an encoder chip for magneto-resistance (MR) linear scales. A typical MR sensor consists of two major parts: one is its base structure, also called the magnetic scale, which is embedded with multiple grid MR electrodes, while another is an “MR reader” stage with magnets inside and moving on the rails of the base. As the stage is in motion, the magnetic interaction between the moving stage and the base causes the variation of the magneto-resistances of the grid electrodes. In this study, a front-end readout IC chip is successfully designed and realized to acquire temporally-varying resistances in electrical signals as the stage is in motions. The acquired signals are in fact sinusoids and co-sinusoids, which are further deciphered by the front-end readout circuit via newly-designed programmable gain amplifiers (PGAs) and analog-to-digital converters (ADCs). The PGA is particularly designed to amplify the signals up to full dynamic ranges and up to 1 MHz. A 12-bit successive approximation register (SAR) ADC for analog-to-digital conversion is designed with linearity performance of ±1 in the least significant bit (LSB) over the input range of 0.5–2.5 V from peak to peak. The chip was fabricated by the Taiwan Semiconductor Manufacturing Company (TSMC) 0.35-micron complementary metal oxide semiconductor (CMOS) technology for verification with a chip size of 6.61 mm^2^, while the power consumption is 56 mW from a 5-V power supply. The measured integral non-linearity (INL) is −0.79–0.95 LSB while the differential non-linearity (DNL) is −0.68–0.72 LSB. The effective number of bits (ENOB) of the designed ADC is validated as 10.86 for converting the input analog signal to digital counterparts. Experimental validation was conducted. A digital decoder is orchestrated to decipher the harmonic outputs from the ADC via interpolation to the position of the moving stage. It was found that the displacement measurement error is within ±15 µm for a measuring range of 10 mm.

## 1. Introduction

The developments of precise measurement and positioning devices have been applied to manufacture processing and testing equipment in the fields of electronic and semiconductor industries. In these devices, linear scale encoders are widely used to detect angular and/or translational positions of the moving part in high-precision machines and robots as the most common position sensors [1,2,3,4,5,6,7,8,9,10,11,12,13]. However, the frequency response of the conventional encoders is limited; thus, they cannot be used in the case of high-speed motions. This problem can be mitigated with a high-resolution linear scale equipped with a high-speed front-end readout and signal processing chip, which can output harmonic signals to obtain greater accuracy and higher resolution. Among different scales, it is known that magneto-resistance (MR) scales, as compared to other scales, especially optical scales [2,3,4,5,6,7,8,9,10], have the advantages of low cost, high speed, accuracy and adequate precision. In comparison of the accuracies offered by MR and optical scales, the optical scale using the reflection principle could achieve a resolution higher than the MR scale, but the MR scale requires much lower costs for components and manufacturing. Much effort was recently made by researchers [4,5,6,7,8] to design successfully and to fabricate an MR scale with a new MR electrode layout and material, aiming to increase the sensing range and resolution of the magnetic scale to the level compatible with the optical scale.

**Figure 1:**
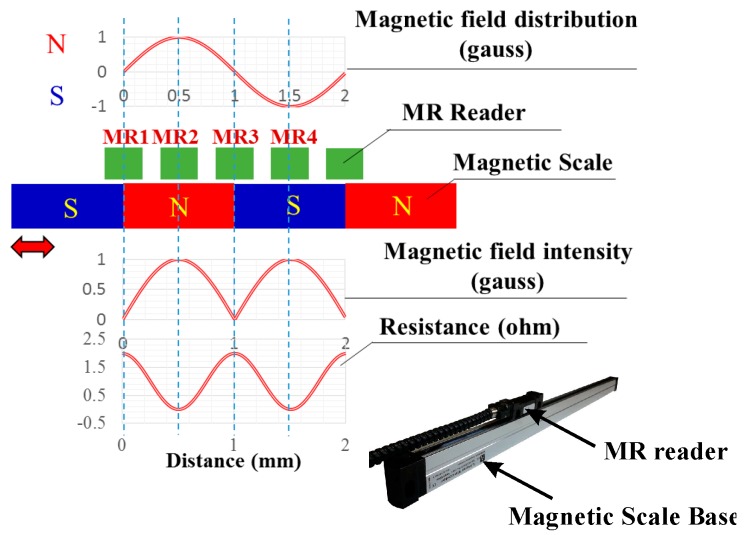
Operation principles of a magnetic linear scale.

**Figure 2:**
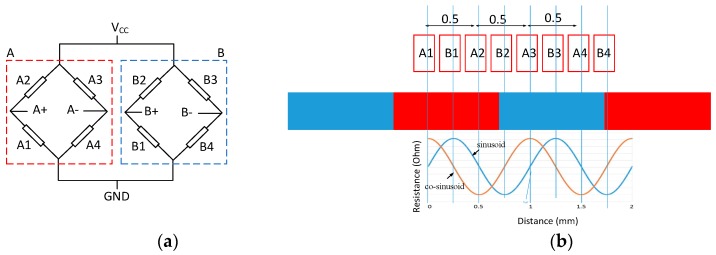
(**a**) Wheatstone bridge; (**b**) electrode layout and resulting harmonic resistance variation.

A typical MR sensor is shown in Figure 1, which consists of two major parts: one is its base structure, also called the magnetic scale, which is embedded with multiple S and N magnets, while another is an “MR reader” stage with multiple MR grid electrodes and moving on the rails of the base. With the stage in motion, the magnetic interaction between the moving reader and the scale base causes the variation of the magneto-resistances of the grid electrodes. The electrodes are designed in a particular way, as shown in Figure 2b, to generate sinusoids and co-sinusoids at outputs A± and B± that are proportional to the resistance variations through a Wheatstone bridge, as shown in Figure 2a. The resulted sinusoids and co-sinusoids in Figure 2b can next be deciphered by a succeeding readout chip to acquire the moving distance of the reader [2,3,4,5,6,7,8,9,10,11]. In fact, in addition to the design of MR electrodes, another critical technology involved in magnetic scales is the readout IC, which needs to be capable of converting the resistance variations of MR electrodes to digits in a high signal-to-noise ratio and then deciphering the resulting digital harmonics to reader positions. Based on the principles of the IC circuit design, the performance of the readout circuit for sensing reader position in real time highly depends on the accuracy and speed of the front-end readout. In the front-end readout, the input harmonic signals from the MR scale need to be amplified to full dynamic range, with zero offsets and no phase error between sinusoids and co-sinusoids [5,6,7,8,9,10,11,12] before entering the analog-to-digital converter ADC for high accuracy. Furthermore, the processing speed of the ADC has to be high enough to cope with the high moving speeds of the reader as opposed to the MR scale base.

In this study, a magneto-resistive (MR) linear scale sensor realized by planar electrodes of MR material is designed for the front-end readout circuit. The MR electrodes in the sensor are connected as in a structure of a Wheatstone bridge, as shown in Figure 2a. Succeeding the MR Wheatstone bridge is a front-end readout circuit proposed to extract the sine/cosine signals from the MR sensor and then converted to digital values by a newly-designed 12-bit hybrid successive approximation register (SAR) ADC. The chip was fabricated by the Taiwan Semiconductor Manufacturing Company —TSMC (Hsinchu, Taiwan) and corresponds to the TSMC 0.35-micron CMOS technology, which was used for verification purposes. A digital decoder is orchestrated and implemented by field programmable gate array (FPGA) codes to decipher the harmonic outputs from the ADC via the technique of interpolation to the actual position of the moving reader stage in real time. It was found that the displacement measurement error is within ±15 µm for a measuring range of 10 mm as compared to the measurement by a precision laser displacement sensor.

The rest of the paper is organized as follows. Section 2 describes the design concept and architecture of the circuit. Section 3 shows the experiment results. Conclusions are given in Section 4.

## 2. Chip Architecture Design

The readout chip for MR sensors proposed in this study consists of two programmable gain amplifiers (PGAs) with common-mode feedbacks for zero DC offset, two 12-bit analog-to-digital converters (ADCs) and a comparator circuit. The readout chip is aimed to extract signals from an MR sensor and then processing it to accurate digital displacement of the moving reader stage in 12 bits.

### 2.1. Programmable Gain Amplifier

**Figure 3:**
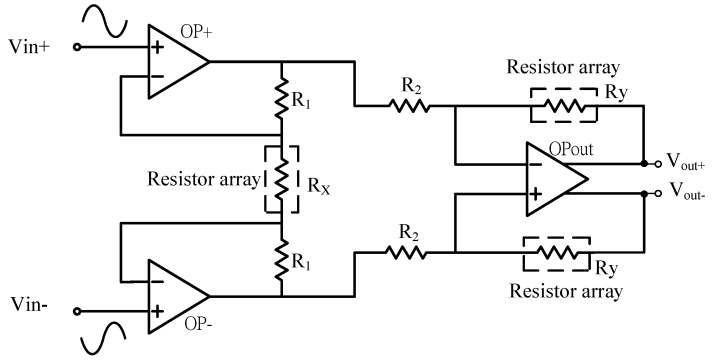
The programmable gain amplifier (PGA).

**Figure 4:**
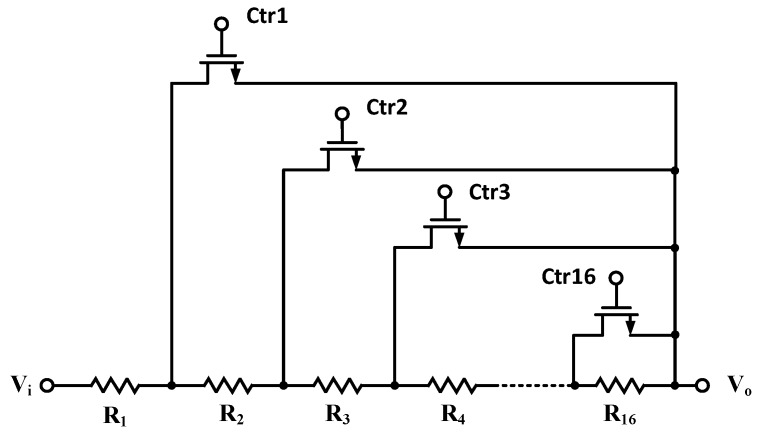
The on-chip switch resistor array in the PGA.

The MR sensor provides two output signals for varying MR resistances. One is sinusoid, while another is the associated co-sinusoids expected to be in a phase difference of 90° relative to the sinusoid. The resistances of the MR sensor are small around 100 kΩ, even with the material of boron mixed in the magneto-resistive material. Thus, in the readout, a programmable gain amplifier (PGA) of input high impedance and adequate gain is first designed [13,14,15,16,17]. The gain of this amplifier is expected to amplify the incoming sinusoids and co-sinusoids to the full dynamic range for the maximum signal-to-noise ratio (SNR) and also zeroing the DC offset. The proposed PGA is shown in Figure 3, which is in a topology of a differential instrumentation amplifier (IA) for large gain tuning ranges. This PGA in fact consists of three operational amplifiers (OPs), which are two identical single-input op-amps and one fully-differential folded cascade. The overall gain of this IA is:

(1) $$\frac{V_{out}}{V_{in}}=(1+\frac{2R_{1}}{R_{X}})\times \frac{R_{y}}{R_{2}}$$

where *R_x_* and *R_y_* are realized by on-chip resistor arrays of MOS switches, as illustrated in Figure 4, to realize tunable gains with the aim to reach full dynamic range at the outputs for sinusoid and the associated co-sinusoids. *R_x_* is tunable in two digital bits and responsible for coarse tuning, while *R_y_* is in four bits and responsible for fine tuning. *R_y_* is automatically adjusted in an on-line fashion based on the output, *V_out_*±, while *R_x_* is tuned externally by two bits given by an external FPGA module. As a result, the differential output voltage is expected to be amplified successfully to full dynamic ranges at *V_out_*±.

**Figure 5:**
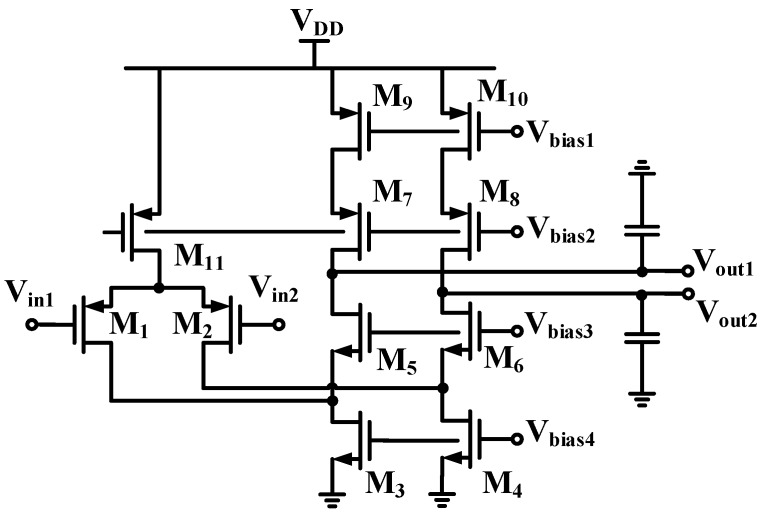
The folded cascade amplifier circuit.

Since the performance of this PGA mainly depends on op-amps, thus, high performance op-amps are required, especially for OP_out_ at the out stage of the IA, in Figure 4. For this OP_out_, a fully-differential folded cascade is proposed and shown in Figure 5. A folded cascade op-amp uses cascading in the output stage combined with a special implementation of differential amplifier to achieve favorable input common-mode input range. This folded cascade op-amp is expected to offer self-compensation, good input common-mode range and a large gain of a two-stage op-amp. To these aims, it is composed of a p-channel differential input pair (M_1_, M_2_), followed by the common-gates stage (M_5_, M_6_) and the current source (M_3_, M_4_). Bias voltages V_bias1_–V_bias4_ are provided to M_3_–M_10_ in order to meet the current specifications at the cascode stage, so as to keep all transistors in the saturation region. The small-signal voltage gain of this circuit is then:

(2) $$A_{V}=G_{m1}R_{o}$$

where *G*_*m*1_ is the overall transconductance of the folded cascade amplifier in Figure 5; *R_o_* is the output resistance of the amplifier, which can be derived as:

(3) $$R_{o}=[(g_{m5}+g_{mb5})r_{o5}(r_{o1}//r_{o3})]//[(g_{m7}+g_{mb})r_{o7}r_{o9})]$$

**Figure 6:**
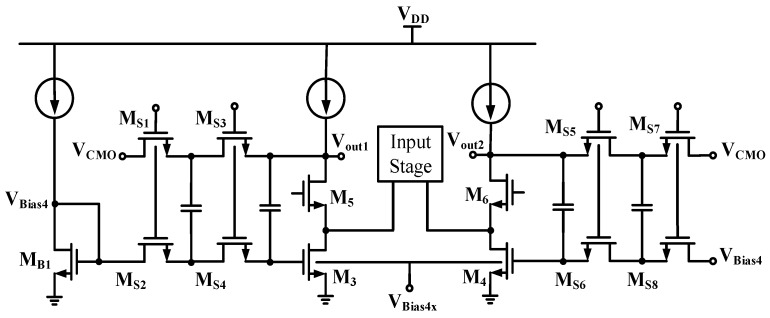
The switched-capacitor common mode feedback circuit.

Despite the previously-designed fully-differential circuit being implemented along with some advantages, the common-mode (CM) loop gain from the external feedback loop around the fully-differential op-amp is quite small, and the CM voltage is not precisely stably defined. Without proper control, the output CM voltage tends to drift to the supply rails due to power supply variations, process variation, offsets, etc. Thus, an additional continuous-time common-mode feedback (CMFB) sub-circuit is applied herein to stabilize the common-mode voltage of the amplifier in Figure 5. Note that the CMFB circuit is utilized in a fully-differential op-amp to keep the op-amp outputs balanced around a known CM voltage V_CM_. In order to reduce power consumption, a switched-capacitor common-mode feedback (SC-CMFB) [18,19,20] in conjunction with the previously-designed folded cascade amplifier in Figure 5 is designed and shown in Figure 6. The input stage, shown in a box, consists of a p-type metal-oxide-semiconductor (PMOS) differential pair with the drains of PMOSs connected to the sources of n-type metal-oxide-semiconductors (NMOSs) (M_5_, M_6_). The rest of the elements in Figure 5 constitute the SC-CMFB circuit. The main advantages of SC-CMFB are that they impose no restrictions on the maximum allowable differential input signals, no additional parasitic poles in the common mode loop and high linearity. For stability consideration, substantial effort has been devoted to ensure fast enough common-mode detection in order to ensure the stability of the whole amplifier. In addition, the CMFB amplifier is designed with adequate gain to make sure good tracking between common mode voltage V_CM_ and the detected common mode voltage at the outputs. Finally, the voltages V_Bias4_ and V_Bias4x_ determine all bias currents for the SC-CMFB circuit.

### 2.2. Switch Resistor Array and Decoder Block Circuit

To facilitate digital gain control for the near full dynamic range at the out of the PGA in Figure 3, variable resistor arrays R_x_ and R_y_’s are implemented using linear resistors in series with the MOSFET switches biased in the triode region, as shown in Figure 3. The variable resistors in the PGA acquire feedback from the analog-to-digital converter (ADC) output stage through digital feedback control to adjust gain and then automatically choose a suitable gain for varying voltage outputs from the considered MR sensor. The PGA, as mentioned in Section 2.2, is facilitated with an internal common-mode feedback (CMFB) circuit to stabilize the output voltage level before the signals reach the subsequent ADC. There are two modes for gain control: fine and coarse tunings. In fine tune mode, a four-bit decoder is designed to specify 16 gain levels for R_y_. On the other hand, a two-bit decoder for coarse tune is adopted for R_x_. The circuit for implementing tunable resistances R_x_ and R_y_’s is shown in Figure 6.

### 2.3. Hysteresis Comparator Design

**Figure 7:**
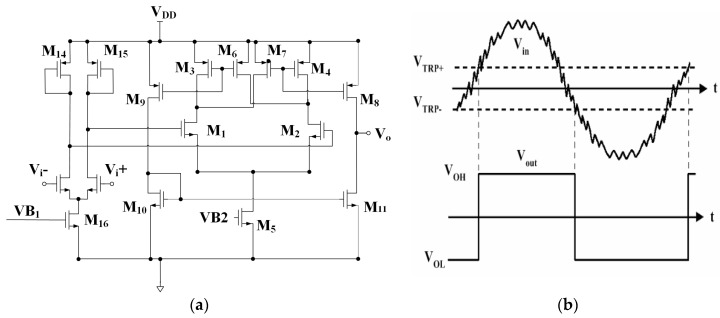
(**a**) The hysteresis comparator with pre-amplifier; (**b**) positive and negative trip voltages.

To generate transistor-transistor logic (TTL) signals for inputting into a digital block circuit to compute succeeding interpolation for deciphering reader stage position, two hysteresis comparator circuits accepting input sinusoids and co-sinusoids from the MR sensor are forged with specially-designed pre-amplifiers inside for substantial noise reduction, both of which are in the same topology as shown in Figure 7a. Hysteresis consideration is designed in many comparators [20,21,22,23]. The inherent hysteresis with pre-designated distinct positive and negative switching voltage levels as illustrated by Figure 7b helps the comparator to avoid ripples at the output due to small amounts of parasitic feedbacks and noises. A two-stage comparator is adopted herein with the first stage as a low-gain pre-amplifier. The input pair MOSs are designed in small sizes and operated in a slow slew rate to minimize kickback noise. The resulting positive and negative switching voltage can be prescribed by the same equation as:

(4) $$V_{TRP\pm }=V_{GS2}-V_{GS1}=\sqrt{\frac{2I_{2}}{\beta_{2}}}+V_{t2}-\sqrt{\frac{2I_{1}}{\beta_{1}}}-V_{t1}$$

In Equation (4), when the differential input voltage (*V_GS_*_2_ − *V_GS_*_1_) is in an increase, M_8_ opens as the current flowing through the MOS M_1_ (i_1_) becomes equal to the current through the MOS M_6_ (i_6_); i.e., as positive trip point voltage (*V_GS_*_2_ − *V_GS_*_1_) ≥ *V_TRP_*_+_. On the other hand, when (*V_GS_*_2_ − *V_GS_*_1_) keeps decreasing and then reaching a negative trip point voltage (*V_TRP_*_−_), the comparator changes state. The current flows through these pairs of the MOSs (M_2_ and M_4_, M_7_ and M_3_, M_6_ and M_1_) are off. 

### 2.4. Successive Approximation Register ADC

**Figure 8:**
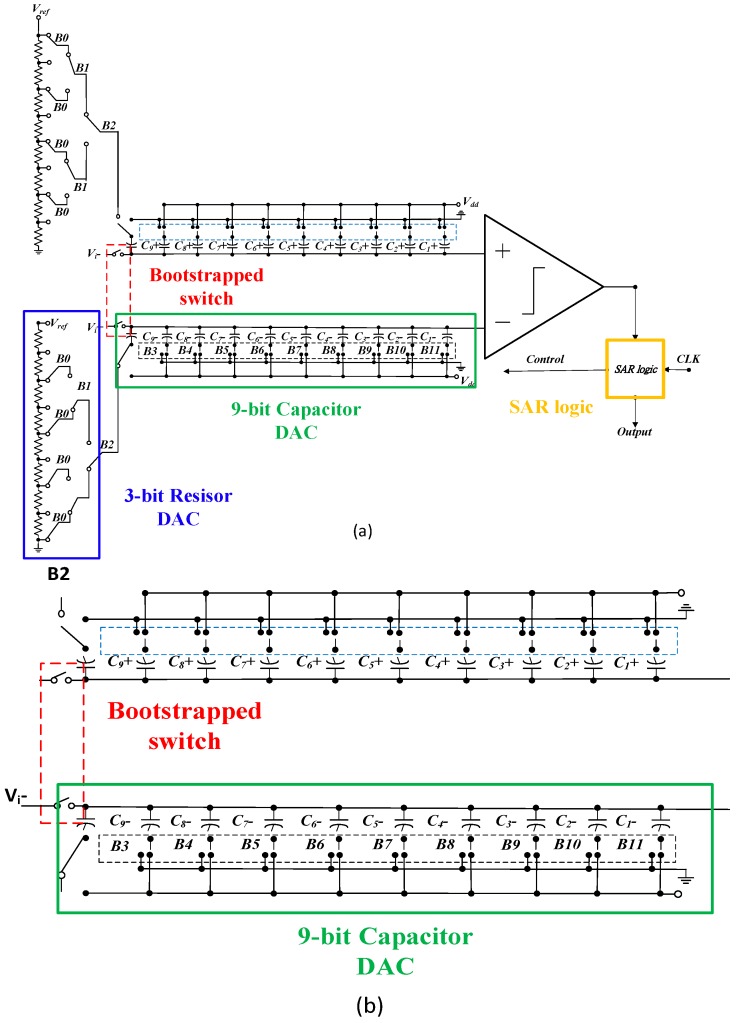
(**a**) The topology of the 12-bit SAR ADC; (**b**) a bootstrapped and partial DAC of the ADC.

**Figure 9:**
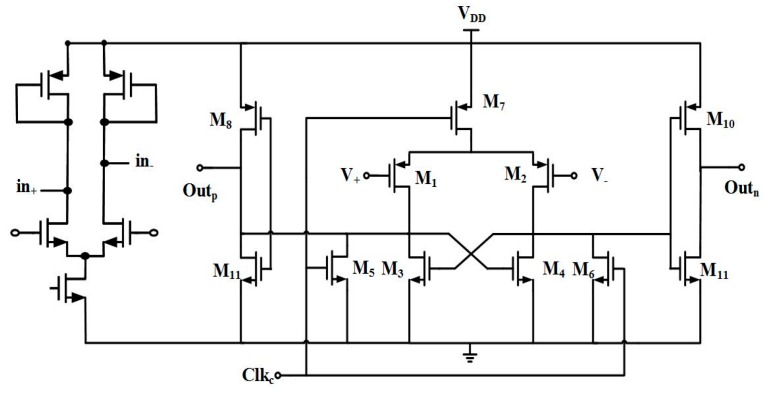
Comparator circuit implemented in the 12-bit hybrid SAR ADC.

The output signal from the PGA is connected to an analog-to-digital (ADC) block to convert analog-to-digital signals before being analyzed in a digital block [21,22,23,24,25,26,27]. The complete topology of the designed 12-bit SAR-ADC, which includes a sample and hold (S/H) circuit, a digital to analog converter, a comparator circuit and a successive approximation register, is shown in Figure 8a. The SAR-ADC design approach is proposed as a hybrid of resistors and capacitors, such that the performance is improved, thus being able to increase the sampling rate and reduce the negative effects of the fabrication process tolerance [25,26,27]. In Figure 8b, the circuits in green and blue blocks constitute the 12-bit digital-to-analog converter, using a binary-weighted capacitor array and resistors in an R-string, whose number of bits is 9 bit and 3 bit, respectively. The unit capacitance used is 10 fF. This adopted architecture owns the advantages of a reduced total capacitance value and small proportion error, while the R-string DAC has high linearity and a low least significant bit (LSB), leading to increasing the resolution significantly. Besides the D/A converter, the comparator has a crucial influence on the overall performance in the SAR ADC. In this study, the topology of the comparator for SAR-ADC is designed and then shown in Figure 9.

This comparator adopts differential inputs of PMOSs to reduce flicker noises and operated on the weak inversion area to increase the highest gain. The PMOSs are connected to the source and drain to avoid body effects. It can also strengthen the input common mode range (ICMR) and the common mode rejection ration (CMRR). When the input *Clk* of the comparator is low, M_3_ and M_4_ are opened, and the comparator is in the comparison state. On the contrary, when the *Clk* is high, M_3_ and M_4_ are closed, and the comparator is in the reset state. The comparator requires high a bandwidth and high gain to increase the switch speed and reduce the return time, but it consumes more power. Thus, an effort has been dedicated herein to achieve a comprise between speed and power consumption.

## 3. Experimental Results

**Figure 10:**
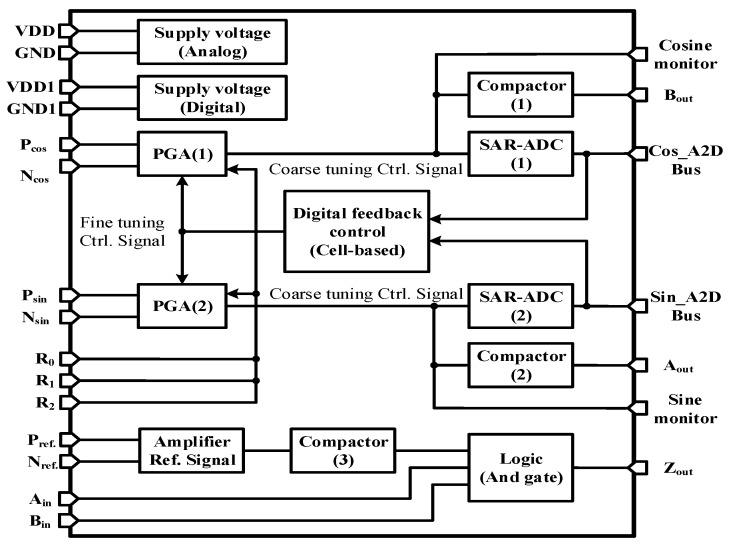
Architecture of the adaptive encoder IC.

**Figure 11:**
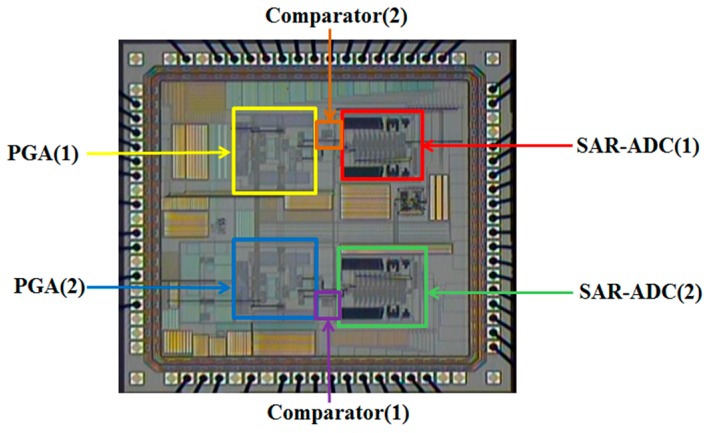
Die photo of the fabricated encoder IC.

Functions in blocks, connections and I/Os of the designed chip are illustrated by Figure 10. With simulations successfully confirming the expected chip performances, the designed chip is fabricated via the TSMC 0.35-µm CMOS process. A die photo of the proposed chip is shown in Figure 11. The chip area is 6.61 mm^2^, while the power consumption is 56 mW. Measurements are conducted for validating the performance of the proposed chip. The sensor output has three different levels of output voltage to the PGA, e.g., 120 mV_p-p,_ 250 mV_p-p_ and 330 mV_p-p_, which are amplified successfully by the designed PGA with 1 mV_p-p_ at the output with adequate precision.

**Figure 12:**
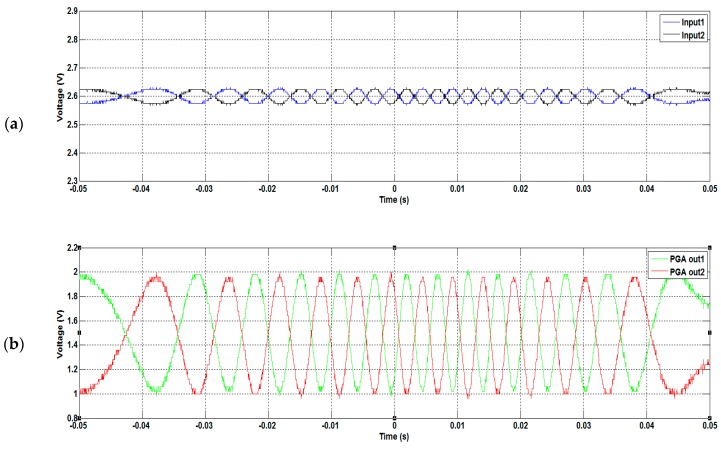
Measured result of the PGA: (**a**) sin/cosine inputs; (**b**) sin/cosine outputs.

**Figure 13:**
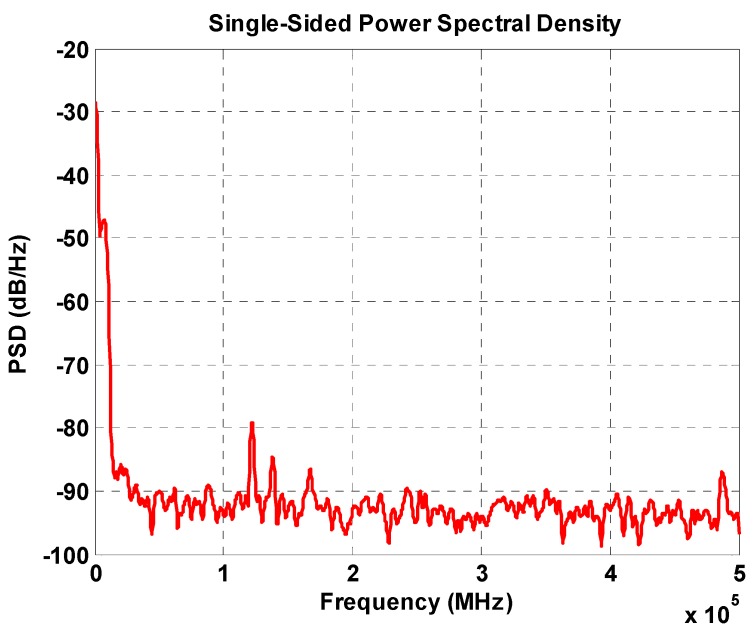
The power spectral density of the PGA output.

**Figure 14:**
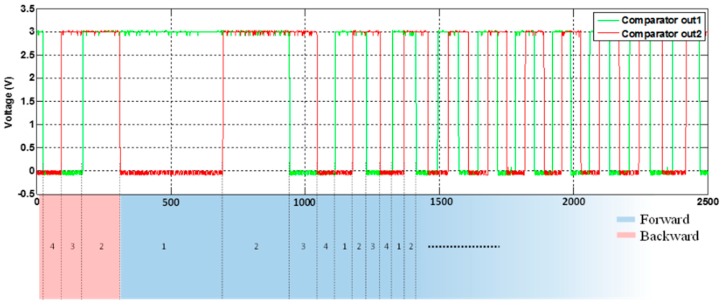
The result of comparator output to determine the direction of the MR sensor.

The measured result of the PGA is shown in Figure 12, where the sinusoids associated with co-sinusoids are amplified with amplitudes reaching to 1 V_p-p_. Figure 13 shows the power spectral density of the PGA output, the noise of which is clearly controlled well below −80.2 dB with a noise floor level under −90 dB/Hz. Following the PGA circuits, two comparators are implemented to generate transistor-transistor logic (TTL) signals for determining the MR reader position, as well as direction. The result of the comparator circuit is shown in Figure 14. Furthermore, it can be seen in Figure 14 that the TTL output is used to determine in which quadrants the MR sensor is. These data are used to specify the director of the MR sensor movement. To compute the position of the sensor, an algorithm was implemented by FPGA to calculate interpolation. The algorithm employed in FPGA is able to determine precise MR reader positions with the capability to compensate the offset and gain errors of the sin/cosine analog signals.

**Figure 15:**
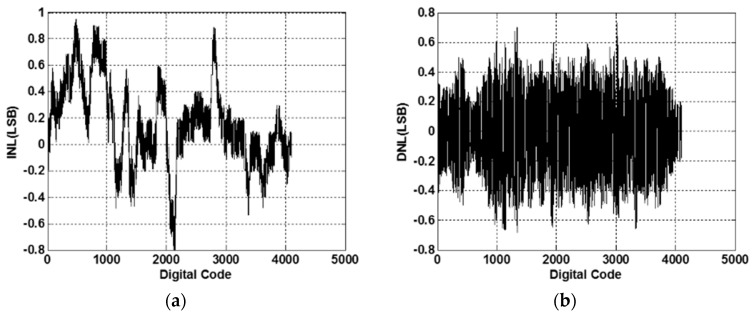
Performance of (**a**) INL and (**b**) DNL of the 12-bit hybrid SAR ADC.

**Figure 16:**
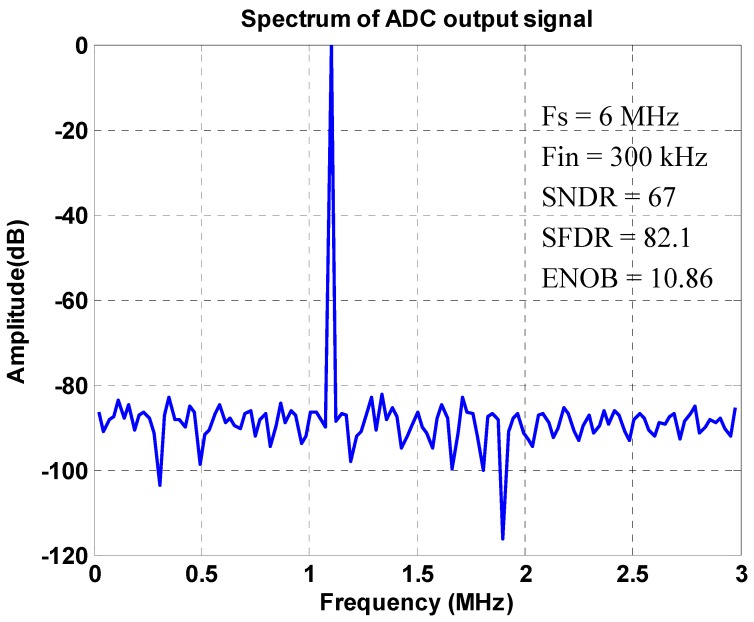
The spectrum analysis result of the 12-bit ADC output.

**Figure 17:**
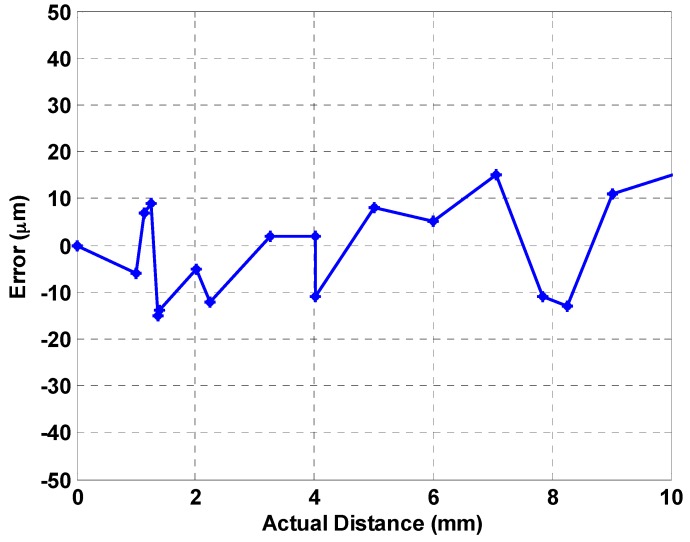
The result of the displacement error in 10 mm.

In addition to the PGA, the designed 12-bit hybrid analog-to-digital converter (ADC) is successfully realized in the tape-out chip for converting the PGA outputs of sinusoids and co-sinusoids in near full-dynamic ranges to digital values in 12-bits. The 12-bit hybrid SAR ADC was designed as stated in Section 2.4, whose sampling rate is up to 5 MHz. It can successfully convert a sinusoidal input with the required precision at a frequency up to 300 kHz. The measured INL is −0.79–0.95 LSB, while the DNL is −0.68–0.72 LSB, as shown in Figure 15, where the linearity of the ADC achieves a good performance (±1 LSB) in input range 1 V_p-p_. The ENOB and all other values to validate the designed 12-bit ADC are shown in Figure 16, where an experimental spectrum on ADC output is calculated and shown. Based on this spectrum, the ENOB is validated as 10.86, which is good enough to convert the input analog signals to digital values for conducting the ensuing interpolation. By utilizing a motor to move the reader stage in the magnetic scale, the readout circuit can successfully extract the output signals from the sensor, and then, the algorithm employed in FPGA computes the position of the sensor precisely. Measurement results show that the position error of the magnetic scale in 10 mm is successfully controlled within ±15 μm, as shown in Figure 17. The summary performance compared to other works is given in Table 1, where it is seen that the present design offers larger interpolation digits in a small chip area and via the relative conventional 0.35-μm process, thus also at low cost.

## 4. Conclusions

A front-end readout circuit is proposed by this study, which can be applied to a magnetic scale for different product specifications. The chip has been successfully designed and fabricated via the TSMC 0.35-µm 2P4M CMOS technology. The chip area is 6.61 mm^2^, while the power consumption is 56 mW under a 5-V power supply. Experiment results show that the PGA successfully amplifies the MR electrode outputs of three different voltage levels, 120 mV_p-p_, 250 mV_p-p_, 330 mV_p-p_, to 1 mV_p-p_ at the output with adequate precision. This is achieved primarily via auto-adjusting by an on-chip tunable feedback resistance to control the PGA gain. The chip is also validated such that the output of the differential signals is stable. As for the designed ADC following the PGA, the measured INL is −0.79–0.95 LSB, while the DNL is −0.68–0.72 LSB. The ENOB of the 12-bit ADC experimentally reaches 10.86, while the linearity of the ADC has been achieved as ±1 LSB in the input range of 0.5–2.5 V. With the above achieved performance of the PGA and ADC, the fabricated readout chip is next connected to a digital interpolation chip for deciphering the MR reader position in realistic digits. Based on the experimental results, the displacement error is validated to be as small as other works, ±15 μm, at a distance of 10 mm in length. Finally, as compared to other similar chip designs and based on the experimental results, the present readout design offers larger precision interpolation digits at the output in a small chip area and via the relatively conventional 0.35-μm process, thus also at low cost.

## Figures and Tables

**Table 1:** Performance comparison with other interpolation methods.

	[5]	[28]	[29]	This Work
Technology	N/A	0.35-μm CMOS	0.5-μm CMOS 2P2M	0.35-μm CMOS 2P4M
Interpolation Method	Advanced Adaptive Phase-locked loop	ADC	Comparator-based	ADC and comparator
Sampling Rate	N/A	100 kS/s	Not needed	600 kS/s
Interpolation Factor	200	-	5, 10, 40 (optional)	1000 (magnetic)
Output Format	Sine signal	Counter code	Pulse stream	Counter code
Chip Area	N/A	1.7 mm^2^	3 mm^2^	6.61 mm^2^
Power	N/A	90 mW	50 mW	56 mW
